# The Therapeutic Role of Dienogest in Endometriosis-Associated Pain: From Basics to Clinics

**DOI:** 10.3390/biom16081080

**Published:** 2026-07-23

**Authors:** Ying Yan, Xi Lan, Huaxi Mai, Hao Tan, Yanchun Liang

**Affiliations:** 1 Department of Obstetrics and Gynecology, Guangxi Hospital Division of the First Affiliated Hospital, Sun Yat-sen University, Nanning 530025, China; 2 Department of Obstetrics and Gynecology, The First Affiliated Hospital of Sun Yat-sen University, Guangzhou 510050, China; 3 Guangdong Provincial Clinical Research Center for Obstetrical and Gynecological Diseases, Guangzhou 510050, China

**Keywords:** endometriosis, endometriosis-associated pain, pathogenesis, hyperalgesia, dienogest, therapeutic effects

## Abstract

The most common symptom of endometriosis is pain. The pathogenesis of endometriosis-associated pain remains unclear. In this review, we illustrate the pathogenesis of endometriosis-associated pain and the mechanisms of dienogest acts on endometriosis-associated pain, as well as therapeutic applications targeting these processes, by searching the literature in PubMed and Web of Science until May 2026. Abnormal distribution of nerve fibers, central sensitization, imbalance of inflammatory and immune environment, and hypoxic stress contribute to the generation of endometriosis-associated pain. Dienogest, as a fourth-generation selective progesterone receptor agonist, plays a key role in the treatment of endometriosis-associated pain by reducing serum estrogen levels, alleviating inflammatory responses, regulating nerve growth factor, modulating control central nervous system allergies, and influencing the biological processes of endometriotic stromal cells. Moreover, its long-term safety and tolerability are relatively satisfactory according to clinical studies, making it an effective therapeutic agent for endometriosis.

## 1. Introduction

Endometriosis (EMS) is a common gynecological benign disease, with pain being one of its most troubling symptoms. Three common types of EMS include ovarian endometrioma, peritoneal EMS, and deep infiltrating endometriosis (DIE) [1]. In addition, there some rare types such as pulmonary EMS, pleural EMS, and abdominal wall EMS. A considerable proportion of ovarian endometrioma patients have atypical pain symptoms and are often diagnosed during routine health examination. DIE is the main cause of different degrees or types of pain in patients. For example, when the vagina or rectovaginal septum is involved, it can cause dyspareunia. Patients with intestinal involvement (mostly the rectum) usually experience tenesmus or defecation pain [2]. Furthermore, if the bladder is infiltrated by the lesion, it can lead to suprapubic pain and other discomforts.

For the treatment of endometriosis-associated pain (EAP), many international guidelines have provided different management strategies [3,4,5,6], and most of the above guidelines recommend progestins as first-line medical treatment for EAP [7]. However, current medical treatment still faces challenging issues such as limited options, significant individual differences, and long-term tolerability. Surgical treatment is also needed to remove the lesions and relieve pain when drugs do not work effectively. Dienogest, a fourth-generation selective progesterone receptor agonist, is one of the crucial drugs in the treatment of EAP [8]. It can centrally inhibit the hypothalamic–pituitary–ovarian (HPO) axis and decreases the serum estrogen level of patients. Moreover, it can act on ectopic endometrial lesions directly, inhibiting angiogenesis, and reducing the inflammatory responses, as well as regulating the immune environment of the lesion. Since it was first applied in China in 2019, it has been widely used in clinical practice. This article aims to synthesize and critically summarize the pathogenesis of EAP, as well as illustrate the underlying mechanism of action of dienogest on EAP and its clinical applications. We conducted a comprehensive literature search in PubMed and Web of Science. The search scope included research articles and reviews published until May 2026 with the keywords “endometriosis”, “endometriosis-associated pain”, “dienogest”, “pathogenesis”, “central sensitization”, and “immune inflammation” in different combinations. Only articles in English were considered. We evaluate the searched literature according to the predefined inclusion criteria. Through this review, we expect to provide a deeper understanding of dienogest in managing EAP, and to promote a wider application in the treatment of EAP.

## 2. Pathogenesis of Endometriosis-Associated Pain

Pain, a subjective feeling, is an uncomfortable sensation or emotional experience that may be caused by actual or potential tissue damage. When peripheral nociceptors receive pain signals, the peripheral nervous system transmits them to the central nervous system, and the brain generates the perception of pain. Medical descriptions of pain include nociceptive pain, inflammatory pain, neuropathic pain, central pain, and hyperalgesia. Different terms have distinct definitions, but we do not use them to describe how the patient feels when we estimate patients in our clinic. Multiple mechanisms have been proposed to play important roles in the generation of pain perception, such as inflammatory reaction, sympathetic and parasympathetic nerve interaction, and especially estrogen action in females [9].

### 2.1. Abnormal Distribution of Nerve Fibers in EMS Lesions

Studies have shown that there is an active crosstalk between endometriotic lesions and nerve fibers. On the one hand, endometriotic lesions can promote excessive innervation, and on the other hand, the abnormally distributed nerve fibers and nerve infiltration contribute to the progression of EMS and EAP [10]. Noxious stimuli caused by damage to non-neural tissues in the lesions can activate visceral nociceptive C fibers, thus generating pain signals. There are various types of nerve fibers in EMS, including Aδ, C, cholinergic, and adrenergic fibers. The imbalance of nerve fiber innervation in lesions is significantly associated with pain generation and severity. On one hand, the density of sensory nerve fibers in lesions is higher than that in adjacent areas. The highly dense sensory nerve receptors can easily generate pain signals upon minor stimulation, sending them to the central nervous system, which indirectly suggests that pain generation depends on nerve fiber density changes. On the other hand, the increased expression of neurotrophic factors (NTFs) in lesions, such as nerve growth factor (NGF), which works by binding to the high affinity receptor tyrosine receptor kinase A (TrkA), promotes neurite growth in NGF responsive neuronal subsets and increases the excitability of nociceptors [11]. Studies also found that the imbalance in sympathetic nerve innervation within and adjacent to the lesions is a potential cause of pain [12]. Semaphorin 3A, a neuronal chemorepellent, serves as a key axon guidance molecule in peritoneal EMS and uterosacral ligament DIE [13]. The density of sympathetic nerves in lesions is lower than that in adjacent areas, and the semaphorin family of nerve inhibitory proteins is an important cause of this imbalance, which was confirmed by resent study [14].

**Figure 1:**
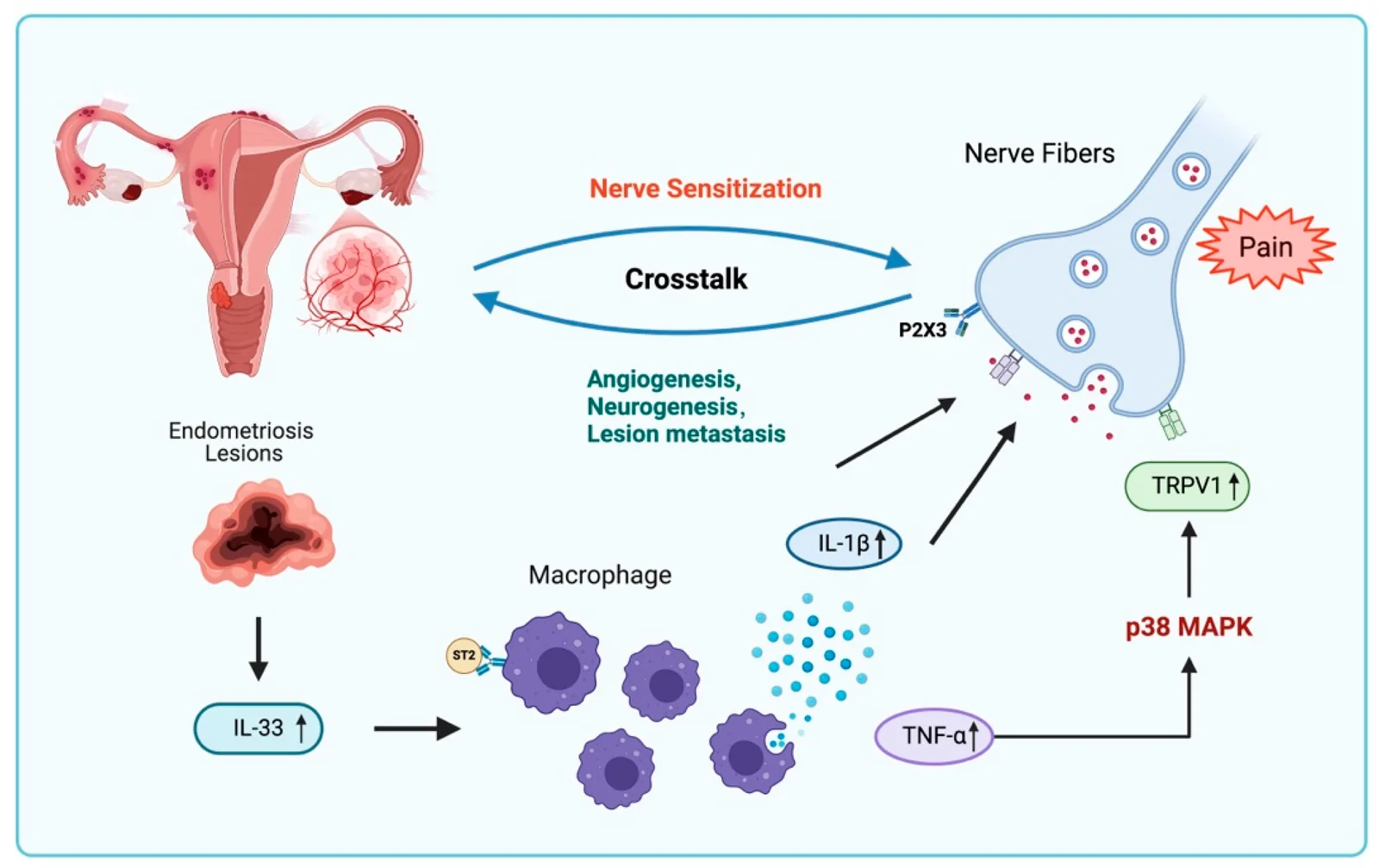
Active crosstalk between endometriotic lesions and nerve fibers. EMS promotes excessive innervation, and abnormal nerve infiltration contributes to angiogenesis, neurogenesis, lesion metastasis of EMS and EAP. Meanwhile, endometriotic lesions alter the immune microenvironment, with IL-33 levels abnormally increasing. IL-33 interacts with the ST2 receptor in macrophages, enhancing the release of TNF-α and IL-1β, triggering an inflammatory cascade and causing allodynia. IL-33 also promotes macrophage aggregation around nerve fibers and increases the density of TRPV1-positive nerve fibers by activating the p38 MAPK pathway. P2X3 and TRPV1 are key receptors in neuropathic pain, which are expressed in abnormally distributed sensory nerve fibers, causing EAP. Created in BioRender. yan, Y. (2026) https://BioRender.com/uyapa0k (accessed on 15 May 2026).

In conclusion, there is a close interaction between nerve fibers and endometriotic lesions by the role of nerve factors, which promotes the development of EMS and induces EAP [10], as shown in Figure 1.

### 2.2. Inflammatory Microenvironmental Changes

The inflammatory microenvironment of EMS lesions leads to an increase in nociceptive signals. The retrograde menstrual blood implanted in the pelvic and abdominal cavities triggers inflammatory responses. The ectopic endometrium undergoes repeated tissue injury and repair, producing a “repair after trauma” biological effect (called repeated tissue injury and repair, ReTIAR), which promotes the formation of lesions and the proliferation, invasion, and metastasis of ectopic endometrium. During this process, both inflammatory cells and cytokines increase to varying degrees, including Tumor Necrosis factor-Alpha (TNF-α), Interleukin 1β (IL-1β), and Monocyte chemoattractant protein-1 (MCP-1). Several studies have found that the concentration of inflammatory cytokines in the peritoneal fluid of EMS patients with pain is higher than that in patients without EMS, indirectly confirming the above theory. Additionally, the increase of specific proteins in the peritoneal fluid also plays an important role. For example, compared with women without EMS, the mRNA concentration of Transient receptor potential cation channel subfamily V member 1 (TRPV1) in the adjacent peritoneal tissue of endometriosis is significantly higher, which contributes to the induction and maintenance of chronic pelvic pain [15,16,17]. During inflammation, phosphorylate TRPV1 increased the sensitivity of injury receptors, thus TRPV1 was considered as a potential target for pain intervention [18]. The elevation of transforming growth factor-β1 (TGF-β1), osteoprotegerin (OPG), and glycodelin in the peritoneal fluid may also be related to the pain intensity of EMS, and their mechanism of pain-generating action is likely through peripheral nerve sensitization [19].

### 2.3. Immune Imbalance

Studies indicate that the formation of endometriotic lesions can trigger abnormal expression of immune related molecules, promoting the release of inflammatory cytokines and causing a cascade effect that leads to neurogenic inflammation [20]. Immune cells associated with EAP include macrophages, neutrophils, and mast cells [21], with macrophage activation being a key contributor to the development of severe EMS through multiple processes such as inflammation, proliferation, angiogenesis, and neurogenesis [22]. It is also an important factor in the development of neuropathic pain. Increased infiltration and degranulation of mast cells have been observed in endometrial tissues from both human and animal models, which amplifies local immune responses. Furthermore, mast cells release IL-6, which activates the JAK-STAT signaling pathway and stimulates the production of prostaglandin E2 (PGE2) and calcitonin gene-related peptide (CGRP), thereby exacerbating inflammatory pain [23,24]. Li et al. [25] have shown that IL-33 promotes the aggregation of macrophages (CD68+) around nerve fibers, thus increasing the density of TRPV1 (+) nerve fibers. Additionally, Ding et al. [26] found that macrophage-derived Netrin-1 was co-expressed with CD68 (a macrophage marker) in endometriotic lesions, and also promoted nerve fiber infiltration in a different way. All the results revealed that immune cell and nerve fiber interaction contributes to the development of EAP. Conversely, macrophage depletion can relieve EAP, as evidenced by reduced spontaneous pain and mechanical hyperalgesia [22]. The mechanism of immune imbalance contributing to EAP is complex. Nerve fiber infiltration, induction of inflammatory cytokine release, and interaction between immune cells and nerve fibers work together to stimulate peripheral nerve sensitization and finally exacerbate EAP (Figure 1).

Furthermore, genetic factors influence the immune–inflammatory crosstalk underlying EMS. Recent genetic research [27] identified 80 genomic regions associated with endometriosis risk by genome-wide association studies (GWAS), and polygenic risk score (PRS) further linked this risk to pathways involved in cell differentiation, immunity and inflammation, hormonal regulation, and tissue remodeling. In addition, Mao X. et al. [28] highlighted a potential link between the function of HRH1 (a genetic susceptibility locus) and pain sensitivity, as well as inflammatory processes associated with endometriosis. However, the precise mechanisms underlying this association remain to be further investigated. A meta-analysis of GWAS [29] revealed that many identified signals were associated with pain perception and maintenance (SRP14/BMF, GDAP1, MLLT10, BSN, and NGF). NGF expression (a GWAS locus) may partly mediate perilesional nerve density around EMS lesions, which is associated with dyspareunia [30].

These studies provide a novel perspective that endometriosis is a genetically susceptible disease, and that susceptibility loci contribute to disease progression and pain generation via multiple pathophysiological pathways. This provides a new direction for future research into EAP pathogenesis and lays a foundation for the discovery of novel therapeutic targets.

### 2.4. Neuropathic Pain and Nervous System Sensitization

When nerve endings are activated by inflammatory reactions caused by the invasion of the ectopic endometrium, sensitization of peripheral nerves of EMS lesions to injury receptors is induced, which is similar to the process of “ReTIAR”. This sensitization is facilitated by a shift from high threshold injury receptors to lower-level receptors. As a result, this transformation can reduce the threshold of ion channels, increase membrane excitability, or enhance receptor expression to mediate sensitization, ultimately aggravating the severity of pain in EMS patients [31]. Therefore, this pathogenesis may have a synergistic effect with the initial process, which contributes to the development of pain. In addition, previous study also found that the fascicular infiltration of nerves in endometriotic lesions exhibited a direct correlation with the severity of pain. Further, nerve injury could stimulate neovascularization in the lesions along with the generation of new nerves. This process is also known as neuroangiogenesis, and may further contribute to the generation of pain [32].

**Figure 2:**
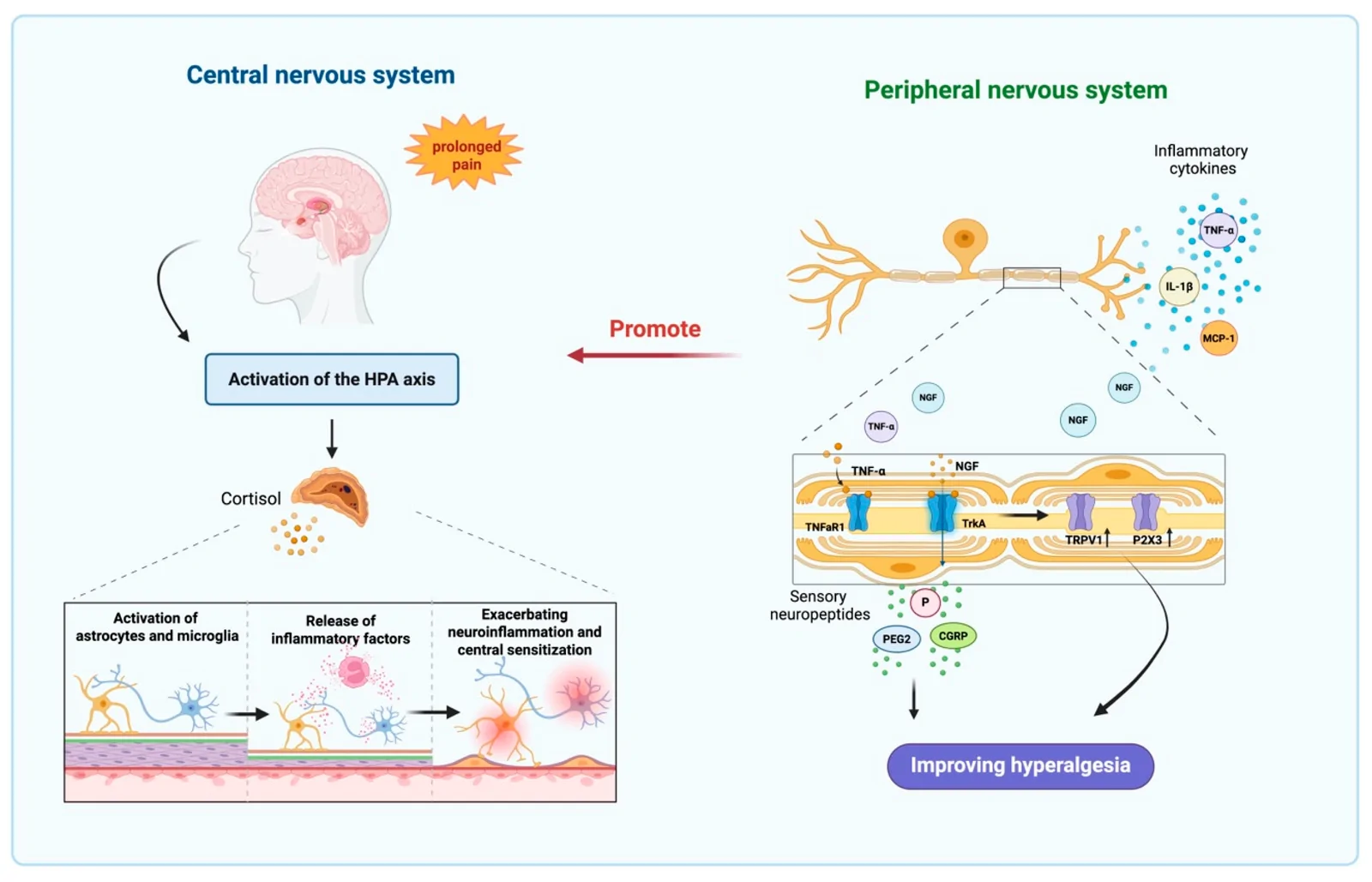
Neurological sensitization mechanisms. **Central sensitization:** The HPA axis triggers continuous cortisol release and causes resistance; cortisol activates microglia and astrocytes via glucocorticoid receptors (GR), releasing more inflammatory factors and ultimately exacerbating neuroinflammation and central nervous system sensitization. **Peripheral nerve sensitization:** NGF directly acts on TrkA receptors of peripheral sensory neurons, raising their excitability and sensitivity, and increasing TRPV1 and P2X3 ion channel expression at the same time, which causes hyperalgesia. NGF also lowers some nociceptor thresholds, stimulating small sensory neurons to release pain-related neuropeptides like Substance P and CGRP, further causing hyperalgesia. Created in BioRender. yan, Y. (2026) https://BioRender.com/tp5z3zk (accessed on 18 May 2026).

The central nervous system (CNS) plays an important role in EAP. There are volume changes in brain regions of EMS patients, as well as alterations in connectivity and altered electroencephalographic activity. Microglia and astrocytes play a crucial role in causing neuroinflammation and promoting central sensitization [33,34]. In addition, prolonged and repeated pain stress activates the hypothalamic–pituitary–adrenal axis (HPA axis), causing cortisol resistance, which further activates microglia and astrocytes and exacerbates neuroinflammation [35]. This mechanism keeps the body in a fight-or-flight response, enhancing pain feedback and central sensitization. Indeed, sensitization of sensory nerves is caused by nociceptors activation. In nociceptors, P2X3 and TRPV1 are key receptors for neuropathic pain [36,37]. Upregulation of TRPV1 and TRPA1 expression has been observed in dorsal root ganglion (DRG) neurons of both humans and rodents, which is associated with enhancing pain sensitivity and estrogen regulation [38,39]. Studies have demonstrated that P2X3 receptors are expressed in stromal and epithelial cells of endometriotic lesions. Additionally, upregulation of P2X3 in dorsal root ganglia (DRG) via the activating transcription factor 3 (ATF3)/activator protein-1(AP-1) pathway contributes to endometriosis-related hyperalgesia [36,40]. Prolonged exposure of sensory nerves to an inflammatory environment may increase central sensitization [31]. NGF can lower nociceptor thresholds, stimulating small sensory neurons to release pain-related neuropeptides, such as Substance P and calcitonin gene-related peptide (CGRP), thus causing hyperalgesia [41]. NGF knockdown can inhibit the growth of endometriotic lesions, significantly reduce nerve fiber density in EMS, and alleviate hyperalgesia [42]. Additionally, pain perception of EMS patients is also influenced by psychological factors like mood, cognition, emotion, and social adaptation, which are processed through the CNS [43] (Figure 2).

### 2.5. Hypoxic Stress Response

When endometrial cells detach from the uterine cavity and move forward into the peritoneal cavity, the microenvironment of the peritoneum suffers from severe hypoxic stress [44]. This hypoxic environment promotes epigenetic regulation in cells, activating various survival processes, including steroidogenesis, angiogenesis, inflammation, and metabolic switching. Hypoxia-inducible factors (HIFs) play a central role in this process, which can drive adaptive changes via transcriptional regulation to help endometriotic cells survive in this harsh microenvironment. Specifically, hypoxia can increase estrogen biosynthesis and then enhance its responsiveness through HIFs, promote angiogenesis, boost cyclooxygenase-2 (COX-2) expression to fuel inflammation, and alter gene expression patterns via epigenetic regulation, all of which facilitate EMS progression [44,45]. TGF-β1, a key growth factor regulating cell proliferation, differentiation, angiogenesis, and immune responses [46], can induce the expression of vascular endothelial growth factor (VEGF) and HIF-1α, thereby promoting angiogenesis in EMS. Studies have found that the mRNA and protein levels of TGF-β1, VEGF, and HIF-1α in EMS tissues are significantly higher than those in normal endometrial tissues [45]. Under hypoxic conditions, the synergistic action of TGF-β1 and HIF-1α leads to a substantial increase in VEGF expression [47], finally promoting neoangiogenesis in EMS. Hence, hypoxic stress is vital for EMS progression, and it may be a key mechanism underlying EAP, though its specific role requires further investigation.

The above research has proved that the pathological changes in EMS involve active crosstalk among nerve fibers, immune system, inflammatory factors, central and peripheral nerve sensitization, and hypoxic stress, which work together to lead to the occurrence of EAP. Therefore, the potential effective drugs for the pain treatment are based on the above mechanisms.

## 3. The Therapeutic Role of Dienogest in Endometriosis-Associated Pain

### 3.1. Mechanism of Action of Dienogest in EAP Management

From drug development to clinical research and market entry, researchers have extensively studied the mechanisms of dienogest in relieving EMS symptoms and controlling disease progression. More and more preclinical studies continue to explore its molecular mechanisms. EMS is an estrogen-dependent chronic inflammatory disease that may induce pain through peripheral–central nervous system crosstalk. Next, we focus on how dienogest acts on the central and peripheral mechanisms to exert the therapeutic effect in EAP treatment and lesion control.

#### 3.1.1. Dienogest Can Alleviate Inflammatory Response in EMS Lesions

Down-regulation of inflammatory factors in lesions is a key therapeutic mechanism of dienogest for EMS. Inflammasomes, especially NOD-like receptor pyrin domain-containing 3 (NLRP3), have been confirmed to play a crucial role in the pathogenesis and progression of EMS. Study has shown that after six months treatment with dienogest, serum levels of NLRP3 and oxidative substances (MDA and ROS) in EMS patients are significantly reduced, while levels of antioxidant substances (SOD, TAC, CAT, and GPX) are significantly increased [48]. Additionally, dienogest treatment significantly increased the level of human leukocyte antigen DR (HLA-DR, an anti-inflammatory factor) in peritoneal fluid and ectopic lesions [49], while TNF-α and IL-1β levels were significantly decreased [50]. Furthermore, dienogest could effectively inhibit inflammation at the cellular level. Experts found that dienogest could reduce prostaglandin E2 (PGE2) by down-regulating aromatase and COX-2 in endometriotic stromal cells (ESCs), which was a key pain-inducing factor [50]. Lipopolysaccharide (LPS) and high-mobility group box 1 (HMGB1) can induce the expression of inflammatory factors (IL-8, IL-6, MCP-1) and TLR4 in endometrial epithelial cells (EECs) and activate NF-κB, thereby promoting the progression of inflammatory microenvironment in ectopic lesions [51]. However, dienogest can block this process, ultimately improving inflammation in EMS lesions and alleviating EAP.

Further evidence indicates that dienogest inhibits C-C motif chemokine ligand 20 expression in EECs induced by IL-1β via the progesterone receptor B (PR-B) [52] and suppress IL-6, IL-8, and MCP-1 in ESCs [53,54], achieving anti-inflammatory therapeutic effect. The above results suggest that dienogest can control lesion progression and alleviate pain by modulating the expression level of pro-inflammatory and anti-inflammatory factors in EMS lesions or peritoneal fluid. However, the above mechanism is currently based on in vitro evidence, and further in vivo studies (including animal experiments and clinical trials) are required to validate its clinical effectiveness.

#### 3.1.2. Dienogest Can Modulate Neurotransmitters Involved in Reducing the Pathogenesis of Neuropathic Pain

Multi-level organizational research has confirmed that dienogest can regulate neurotransmitters involved in the pathogenesis of neuropathic pain. Research has indicated that dienogest effectively combats EAP and disease progression by specifically activating PR-A/PR-B to down-regulate the mRNA expression of COX- 2, PGE2, NGF, and VEGF in endometriotic cells [55]. CGRP is a neurotransmitter involved in neuropathic pain. It transmits pain signals, promotes neuroinflammation, regulates NGF expression, and induces abnormal nerve distribution, thereby contributing to neuropathic pain. Studies have revealed that postmenopausal women have significantly lower serum CGRP levels, which hormone replacement therapy can restore to reproductive age levels, indicating CGRP is regulated by sex hormones [56]. Shahla Chaichian et al. [57] found that after six months of dienogest 2 mg qd treatment in EMS patients with pain, serum CGRP levels (69.66 ± 11.53 vs. 80.53 ± 16.13, *p* < 0.05) and pain levels (1.00 ± 2.00 vs. 7.93 ± 1.50, *p* < 0.05) were both significantly decreased. This result suggested dienogest could reduce CGRP to alleviate inflammatory responses and pain in EMS lesions. Researchers also speculate that dienogest may lower estrogen levels by inhibiting the HPO axis, thereby reducing CGRP levels. At the level of animal tissue study, similar conclusions have also been drawn. Using monkeys as an animal model to explore dienogest’s central mechanism in alleviating EAP, it was found that eight weeks of dienogest treatment reduced abdominal hypersensitivity. Functional MRI showed decreased insular and thalamic activation after treatment [58], indicating dienogest can relieve pain symptoms in EMS patients by modulating CNS neurosensitization. Dienogest exerts its significant therapeutic effects both in the central and peripheral systems, which alleviates EAP by regulating neurotransmitters and nerve growth factors.

#### 3.1.3. Dienogest Can Inhibit Cell Proliferation and Promote Apoptosis

Choi et al. [59] proposed that EMS might result from progesterone resistance-induced endoplasmic reticulum (ER) stress, and that dienogest may exert anti-endometriotic effects by modulating ER stress, increasing apoptosis, and inhibiting cell proliferation and invasion. This theory was also supported by a 3D endometrial cell culture model study [60]. In a study focused on the effect of dienogest on cellular biological behavior, dienogest could arrest ESCs in the G0/G1 phase [61] or downregulate cyclin D1 [62], thereby inhibiting cell proliferation. Dienogest also suppressed the activity and proliferation of ESCs induced by the level of estrogen, TNF-α-, IL-1β, and IL-32, and possibly by reducing proliferating cell nuclear antigen (PCNA) expression [50]. It has been confirmed that midkine (MK) can promote cell proliferation, migration, angiogenesis, and fibrosis in EMS. MK is also an important regulatory factor that affects peripheral nerve stimulation and sensitization. The dienogest-mediated reduction in MK expression could underlie its effectiveness in treating EAP [31]. Furthermore, multiple animal experiments demonstrated that dienogest (at 0.3 mg/kg/day, 28 days) significantly reduced ectopic lesions [50,63], suppressed neovascularization (included reducing capillary network area and density), and altered hemodynamics, accompanied by reduced vascular α-smooth muscle actin expression [64]. Dienogest reduces inflammatory factors by inhibiting ESCs proliferation and invasion, which further decreases peripheral nerve stimulation and sensitization, ultimately alleviating EAP.

#### 3.1.4. Dienogest Can Improve the Hypoxic Environment

A key HIFs downstream factor, Yes-associated protein 1 (YAP1) and its downstream miR-21-5p were significantly increased in the hypoxic microenvironment of ectopic lesions. Lin et al. [65] found that dienogest not only reduced serum extracellular vesicle-related miR-21-5p levels in EMS patients but also downregulated YAP1 and miR-21-5p in ESCs and mouse models, thus inhibiting lesion growth and improving the hypoxic microenvironment.

**Figure 3:**
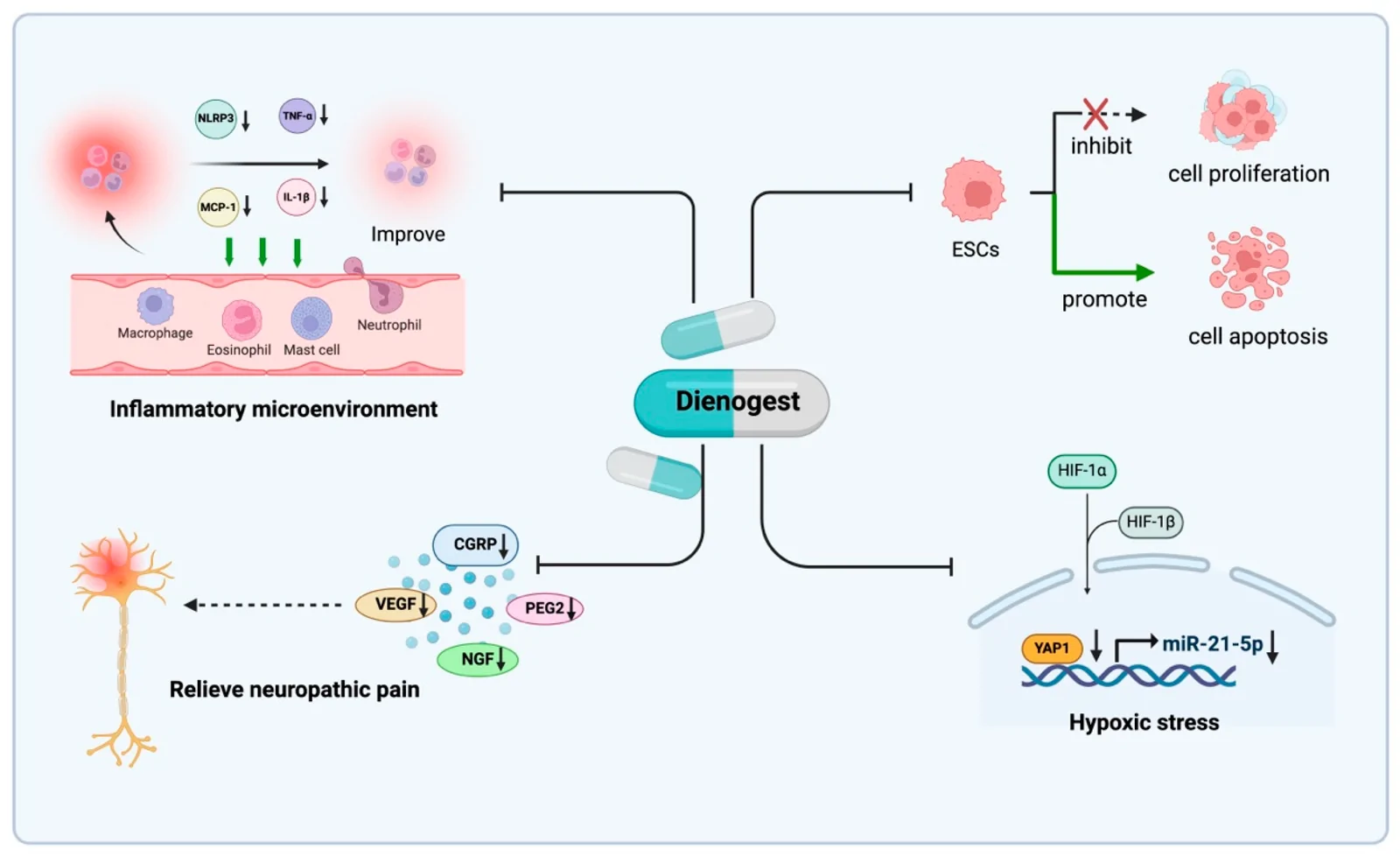
The pathogenesis of dienogest in Endometriosis-associated pain. **Inflammatory microenvironment:** Dienogest controls the progression of the lesion and alleviates pain by reducing the expression of inflammatory factors including NLRP3, TNF-α, and IL-1β. **Relieve neuropathic pain**: By downregulating the expression of CGRP, which is associated with neuropathic pain, as well as NGF, VEGF, and PEG2, dienogest effectively relieves EMS-associated pain and controls disease progression. **Regulating the cell cycle**: Dienogest affects cell biological behavior by inhibiting endoplasmic reticulum stress, thus inhibiting cell proliferation and inducing apoptosis. **Hypoxic stress**: In hypoxic conditions, dienogest regulates HIF downstream factors YAP1 and its target miR-21-5p, thereby effectively improving the hypoxic microenvironment and relieving pain. Created in BioRender. yan, Y. (2026) https://BioRender.com/wtyqq6p (accessed on 22 May 2026).

In summary, dienogest exerts anti-inflammatory effects on endometriotic lesions. However, whether this effect is hormone-dependent or PR-dependent remains to be investigated. Current evidence sufficiently proves that dienogest modulates inflammatory cytokines and neurotransmitters and affects biological processes in endometriotic cells, further altering the lesional microenvironment and nerve distribution, ultimately achieving the effect of controlling EMS progression and relieving pain (Figure 3).

### 3.2. Clinical Applications of Dienogest in EAP Treatment

Based on the above basic studies, dienogest plays a significant therapeutic role in EMS and EAP by molecular pathways. Next, we review and analyze the clinical applications of dienogest in EAP treatment, in order to provide a direction for gynecologists in clinical practice.

#### 3.2.1. Effective Pain Relief and Reduction of EMS Lesions

Techatraisak et al. [66] followed up a prospective study of 865 Asian EMS patients, and found that after 6 months of treatment with dienogest, the scores of all EHP-30 domains were significantly reduced, especially in the “pain” domain, with an improvement rate of 78.4% and an average decrease of 4.5 points in NRS scores. The adverse events reported in the study included amenorrhea, metrorrhagia, vaginal hemorrhage, irregular menstruation, headache, acne, alopecia, and weight gain (sorted by occurrence rate), most of which were of mild-to-moderate intensity. Similar results were confirmed in other studies on the impact of dienogest on the quality of life and pain relief of EMS patients [67,68,69].

Uludag et al. [70] conducted a prospective cohort study and found that after 6 months of treatment with dienogest, the volume of ovarian cysts in patients was reduced by 41% [(112.63 ± 161.31) cm^3^ vs. (65.47 ± 95.69) cm^3^, *p* = 0.005]. The visual analogue scale (VAS) scores for dysmenorrhea, dyspareunia, and chronic pelvic pain were reduced by 35.5% (*p* < 0.001), 37.5% (*p* < 0.001), and 38.5% (*p* < 0.001), respectively.

Muzii et al. [71] conducted a prospective study to evaluate the effect of dienogest on ovarian EMS cyst diameter and EAP, involving 32 patients with unilateral ovarian EMS and pelvic pain. After continuous treatment with oral dienogest (2 mg, once daily) for 6 months, the mean cyst diameter decreased from (4.0 ± 1.3 cm) at baseline to (2.4 ± 1.2 cm) (*p* < 0.0001), with a 40% reduction in diameter and a 79% reduction in volume. Another prospective study [72] also found that after 6 months of treatment with dienogest, the mean cyst volume was reduced by 66.71%, and after 12 months of treatment, it was reduced by 76.19%. The VAS scores for dysmenorrhea decreased by 74.05–96.55% at 6–12 months, while those for dyspareunia and chronic pelvic pain decreased by 42.71% and 48.91% at 6 months, and by 51.93% and 59.96% at 12 months (The data was shown in Supplementary Table S1).

The above studies showed that dienogest has significant and stable effects in relieving dysmenorrhea, dyspareunia, chronic pelvic pain, reduced ovarian endometrioma cysts, and improves quality of life.

#### 3.2.2. Applications of Dienogest in Non-Surgery and Postoperative Management

Chen et al. [73] conducted a retrospective cohort study by dividing EMS patients into a surgery group and non-surgery group to explore pain-relieving effects of dienogest in distinct patient groups. The results revealed that the VAS score was declined from 47.5 mm to 9.6 mm in the surgery group (*p* < 0.05) at 3 months, and further decreased to 2.1 mm at 12 months, respectively. Significant effects were also achieved in the non-surgery group: the VAS score was decreased significantly from 65.7 mm to 13.2 mm at 3 months (*p* < 0.01), and remained at 0.3 mm at 9 and 12 months. Ovarian endometriomas and CA-125 levels were both decreased significantly in the two groups (*p* < 0.01), indicating that dienogest was effective in relieving pain whether in non-surgery or postoperative patients. Further studies confirmed that dienogest relieved pain and protected ovarian function while shrinking ovarian endometriomas [71,74] (Supplementary Table S1).

In EMS postoperative management, dienogest also has significant advantages in relieving pain and preventing recurrence. A prospective study [75] received 146 women who had laparoscopic bowel and parametrial surgery for DIE. They were treated with dienogest and GnRHa after surgery, respectively. They found that both dienogest and GnRH agonists were associated with a highly significant reduction of pain (*p* < 0.001), and a more satisfactory profile and lower recurrence rate was reported with dienogest. Similar results were found in another study [76]. Ying Ma et al. [77] discovered that compared with monotherapy, GnRH-a (3–4 cycles) combined with DNG as postoperative treatment significantly reduced bleeding symptoms (including frequent, irregular, and early adverse bleeding), lowered EHP-5 scores (*p* < 0.05), improved patient compliance, and enhanced overall quality of life. This study indicated that GnRH-a combined with dienogest can prevent endometrial proliferation, reduce adverse bleeding, attenuate estrogen decline, and alleviate osteoporosis. A randomized double-blind study [78] compared the effects of dienogest with Compound oral contraceptive pills (COCP) for pain relief and quality of life in patients with severe endometriosis after laparoscopic surgery. The results showed that both treatments significantly relieved pelvic pain, dyspareunia, and improved quality of life compared with placebo. However, dienogest was associated with lower occurrence of hair loss, headache, hot flashes, and numbness. Another study [79] suggested that dienogest is superior to OCPs in improving pain and QoL in patients with endometriosis, but not as effective as OCPs in pelvic pain and dyspareunia, as the safety was similar between these two drugs. A meta-analysis [80,81] indicated that dienogest maintenance therapy significantly reduced VAS scores at 12 months after EMS surgery and had a lower recurrence rate than LNG-IUS and GnRH- a therapy (Supplementary Table S2).

These studies indicate that dienogest, whether as monotherapy or combined with GnRH a, offers significant benefits in postoperative EMS management. It outperforms GnRH-a, LNG-IUS, and COCP in reducing pain (VAS) and recurrence rates, while demonstrating a more favorable safety profile with fewer side effects, better patient compliance, and improved quality of life. However, most study outcomes occur primarily at 6–12 months post-surgery. This limited timeframe precludes adequate assessment of long-term recurrence patterns, bone mineral density changes, and the durability of the therapeutic effect. Current evidence also faces the challenge of heterogeneous patient populations and lack of objective safety parameters which need further large-scale, long-term prospective studies with standardized outcomes to confirm its superiority.

#### 3.2.3. Applications of Dienogest in the Treatment of Die

DIE is characterized by multiple-site and deep infiltrative lesions, often involving adjacent organs and causing complications such as hydronephrosis and hematuria. These features significantly increase surgical difficulty and elevate the risk of perioperative complications. Recently, a series of clinical studies have proved that dienogest has a significant effect on relieving DIE related pain and shrinking the deep endometriotic lesions [82,83,84,85]. Wu et al. [86] conducted a meta-analyses of 256 patients with DIE, assessing dienogest’s long-term conservative treatment effectiveness over 12 to 36 months. Significant improvements were found in dysmenorrhea (MD = 4.24, 95%CI: 2.92–5.56, *p* < 0.00001), non-menstrual pelvic pain (MD = 3.11, 95%CI: 3.34–3.88, *p* < 0.0001), dyspareunia (MD = 1.93, 95%CI: 1.50–2.37, *p* < 0.00001), and rectosigmoid nodule size (MD = 0.32, 95%CI: 0.18–0.46, *p* < 0.00001). Another prospective cohort study showed that after 12 months of dienogest treatment, patients with DIE had significant improvements in dysmenorrhea, dyspareunia, pelvic pain VAS scores (*p* < 0.05), and female sexual function index (17.6 vs. 22.1, *p* = 0.0023) [87]. The characteristics of the above studies are shown in Supplementary Table S1.

#### 3.2.4. Long-Term Efficacy, Side Effects, and Safety Evaluation

As shown in the above research, dienogest can effectively reduce the volume of ovarian endometriomas and relieve pain, improving patients’ quality of life. However, long-term dienogest treatment still needs to be evaluated for efficacy, side effects, and safety.

In a 108-month observational study [88], the mean diameter of endometriotic cysts in patients with EMS decreased from 33.2 mm (29.4–36.9) to 7 mm (0–15.8) after 108 months of dienogest treatment. At 12 months, VAS scores for dysmenorrhea, dyspareunia, pain during defecation, and non-cyclic pelvic pain significantly improved with treatment, with sustained effects long-term.

Lee et al. [89] conducted a retrospective cohort study of 514 women with ovarian endometriomas from seven hospitals, who received dienogest 2 mg/day for 48–164 weeks after surgery, to assess its long-term efficacy, safety, and recurrence rate. The results showed that dienogest was effective for the prevention of endometriosis pain recurrence after about 6 months of use, and efficacy was sustained for at least 18 months. During long-term dienogest therapy, the discontinuation rate attributed to adverse events was 11.4%, and the most common adverse events were amenorrhea (66.2%) and abnormal uterine bleeding (14.6%). However, in this study, bone mineral density (BMD) was measured in only a small number of participants (12 of 514), the limited sample precluded a thorough assessment of dienogest’s impact on bone loss.

Another study [90] also showed that after 3 months of dienogest treatment, all patients’ dysmenorrhea symptoms disappeared, with VAS scores dropping to 0 mm and remaining so until the end of the study. After 12 months of treatment, ovarian endometriomas were significantly reduced (with a volume reduction rate of 77.62%, *p* < 0.05), CA-125 and CA-199 levels were also significantly decreased (*p* < 0.05). Finally, a larger prospective cohort study in South Korea included 3356 patients with EMS from 73 centers to evaluate the safety and efficacy of dienogest treatment. Despite adverse events including abnormal uterine bleeding, weight gain, headache, and breast discomfort reported during long-term dienogest treatment, 75.51% of participants were satisfied with the treatment outcomes. This large cohort study confirms that dienogest treatment could significantly relieve EAP, with favorable safety and effectiveness [91].

As for the side effects, Atlihan et al. [92] reported that in patients receiving dienogest treatment (2 mg/day), the decreased libido in the long-term group (>12 months) was 4.3 times higher than that in the short-term group (<2 months). Maiorana et al. [88] divided the patients into short-term therapy (<15 months) and long-term therapy (>15 months) groups to assess the effects of dienogest on pain variation. Among all patients, menstrual alterations was the most common adverse effect (22.9%). Compared with the short-term group, the long-term group had significantly higher rates of headache (3.6% vs. 24.7%), weight gain (5.3% vs. 24.7%), and decreased libido (10.7% vs. 45%), *p* < 0.05. The potential mechanism may be its potent progestogen activity; dienogest may cause excessive atrophy of local tissue and exacerbate the discomfort of sexual intercourse [93] (Supplementary Table S1).

Although long-term use of dienogest brought side effects such as decreased libido, weight gain, and spotting, the significant pain relief and reduction in ovarian endometriotic cysts it provides lead to markedly improved quality of patient life, making these adverse effects acceptable to patients.

Dienogest is superior to other agents in relieving pain and improving quality of life, yet it carries unavoidable adverse effects including abnormal uterine bleeding (such as spotting, menstrual irregularities), amenorrhea, weight gain, and so on. The primary limitation of the studies reviewed is their single-arm design without appropriate controls, which prevents attributing all effects on quality of life and safety solely to dienogest and may underestimate confounding factors. Additionally, patient attrition during follow-up may introduce bias into the final efficacy estimates. Thus, the efficacy and safety of dienogest can only be interpreted within a limited scope. In further studies, better-controlled variables and larger sample sizes are warranted to obtain more robust evidence.

In conclusion, dienogest represents a promising medication for the long-term management of endometriosis, especially in patients with severe pain, with acceptable efficacy and safety.

## 4. Conclusions

The pathogenesis of EAP is complex. EAP is the result of multiple and serial effects caused by the initiation of primary ectopic lesions. The ectopic stromal and endometrial cells are influenced by abnormally elevated estrogen, further increasing inflammatory cell counts, cytokine secretion, and causing damage to nerve fibers and their misdistribution. These changes create a microenvironment that not only promotes further lesion proliferation and metastasis but also triggers immune-neuro-inflammatory crosstalk which ultimately causes pain. Moreover, the CNS, as a central sensor and regulator, also plays a key role in the disease progression.

Endometriosis is an estrogen-dependent disease. As mentioned above, estrogen participates in the formation and progression of EMS lesions, and progestin is an appropriate option to prevent the proliferation of estrogen-induced lesions and relieve EAP. Dienogest, as one of the progestins for the treatment of EMS, has a strong progestogenic effect, moderate estrogen suppressing effect, anti-inflammatory, antiproliferative, and antiangiogenic properties. The therapeutic advantage of dienogest may be due to its mild yet sustained anti-estrogenic and analgesic effects, particularly in suppressing endometriotic lesions and alleviating dysmenorrhea, thereby directly improving patients’ physical activity and quality of life.

Dienogest exerts multi-level therapeutic effects on EAP, which involve multiple systems. It could modulate the expression level of neurotrophic factors, pro-inflammatory and anti-inflammatory factors, simultaneously acting on both in the central and peripheral systems to attenuate neural sensitization and ameliorate hypoxia, thereby alleviating pain. Although long-term dienogest treatment may cause adverse events, its tolerability and safety are acceptable within a certain clinical scope. Further basic studies and large-scale clinical studies are required to validate dienogest’s pathogenesis, efficacy, and safety in endometriosis and EAP, along with reducing confounding factors and ensuring high-quality follow-up, for its wider application and promotion.

## Data Availability

No new data were created or analyzed in this study. Data sharing is not applicable to this article.

## Supplementary Materials

The following supporting information can be downloaded at: https://www.mdpi.com/article/10.3390/biom16081080/s1, Supplementary Table S1: General characteristics of the clinical studies and Supplementary Table S2: Dienogest VS. other drugs in EMS postoperative management were showed in the Supplementary file.

## Abbreviations

EMS	endometriosis
EAP	endometriosis-associated pain
DIE	deep infiltrating endometriosis
HPO	hypothalamic-pituitary-ovarian
NTFs	neurotrophic factors
NGF	nerve growth factor
TrkA	tyrosine receptor kinase A
ReTIAR	repeated tissue injury and repair
TNF-α	Tumor Necrosis factor-Alpha
IL-1β	Interleukin 1β
MCP-1	Monocyte chemoattractant protein-1
TRPV1	Transient receptor potential cation channel subfamily V member 1
TGF-β1	transforming growth factor-β1
OPG	osteoprotegerin
CNS	central nervous system
HPA axis	hypothalamic-pituitary-adrenal axis
ATF3	activating transcription factor 3
AP-1	activator protein-1
CGRP	calcitonin gene-related peptide
GWAS	genome-wide association studies
PRS	polygenic risk score
HIFs	Hypoxia-inducible factors
COX-2	cyclooxygenase-2
VEGF	vascular endothelial growth factor
DRG	dorsal root ganglia
GR	glucocorticoid receptors
NLRP3	NOD-like receptor pyrin domain-containing 3
HLA-DR	human leukocyte antigen DR
EECs	endometrial epithelial cells
ESCs	endometriotic stromal cells
PGE2	prostaglandin E2
LPS	Lipopolysaccharide
HMGB1	high-mobility group box 1
PR-B	progesterone receptor B
ER	endoplasmic reticulum
PCNA	proliferating cell nuclear antigen
MK	midkine
YAP1	Yes-associated protein 1
VAS	visual analogue scale
COCP	Compound oral contraceptive pills
Abbreviations in Supplementary Tables	
EHP-30	Endometriosis Health Profile-30
FSDS	Female Sexual Distress Scale
The SF-36	The Short Form-36
AFC	antral follicle count
AMH	antiMullerian hormone
OC	Oral contraceptive
FSFI	Female sexual function index
ISS	Index of sexual satisfaction
GIQLI	Gastrointestinal Quality of Life Index
NRS	The Numerical Rating Scale
SQOL-F	Sexual Quality of Life-Female questionnaire
B-PFSF	Brief Profile of Female Sexual Function
ADR	adverse drug reaction
COCP	Compound oral contraceptive pills
DNG	Dienogest
EHP-5	Endometriosis Health Profile-5
